# The centrosome – diverse functions in fertilization and development across species

**DOI:** 10.1242/jcs.261387

**Published:** 2023-12-01

**Authors:** Abrar Aljiboury, Heidi Hehnly

**Affiliations:** ^1^ Syracuse University, Department of Biology, 107 College Place, Syracuse, NY 13244, USA; ^2^ Syracuse University, BioInspired Institute, Syracuse, NY 13244, USA

**Keywords:** Centriol appendages, Centrioles, Centrosome, Cilia, Pericentriolar matrix

## Abstract

The centrosome is a non-membrane-bound organelle that is conserved across most animal cells and serves various functions throughout the cell cycle. In dividing cells, the centrosome is known as the spindle pole and nucleates a robust microtubule spindle to separate genetic material equally into two daughter cells. In non-dividing cells, the mother centriole, a substructure of the centrosome, matures into a basal body and nucleates cilia, which acts as a signal-transducing antenna. The functions of centrosomes and their substructures are important for embryonic development and have been studied extensively using *in vitro* mammalian cell culture or *in vivo* using invertebrate models. However, there are considerable differences in the composition and functions of centrosomes during different aspects of vertebrate development, and these are less studied. In this Review, we discuss the roles played by centrosomes, highlighting conserved and divergent features across species, particularly during fertilization and embryonic development.

## Teaser

**Summary:** Delving into centrosomes, this Review discusses their versatile roles in animal cells, emphasizing their diverse functions during fertilization and embryonic development across species.

## Introduction

**Fig. 1.**
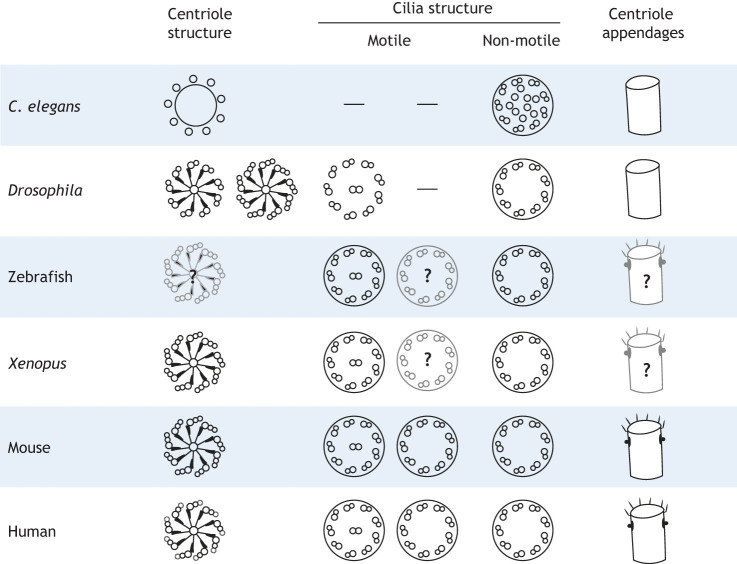
**Divergence in centrosome substructures across model species.** Centrioles have a conserved nine-fold symmetry but can be composed of microtubule singlets, doublets or triplets. *Drosophila* centriole structures can differ based on tissue types, and some tissues have centrioles with both microtubule doublets and triplets. *C. elegans* lack the centriole cartwheel that can be found in other model species. Both motile and non-motile cilia can have a 9+0 and 9+2 structure, which can differ based on the tissue type. However, 9+2 cilia generally tend to be motile, whereas 9+0 are mostly non-motile. Some species have centrioles that are decorated with distal and subdistal appendages at the distal end of the mother centriole. When present, distal appendages have a nine-fold symmetry, whereas subdistal appendages can be present in varying numbers and organization. Gray structures and question marks represent uncertainty in the presence or features of these structures due to a lack of research in that area.

The centrosome consists of a pair of highly ordered barrel-shaped structures, the centrioles, that are embedded in a network of proteins known as the pericentriolar matrix (PCM) (Robbins et al., 1968). Centriole barrels present with nine-fold radial symmetry, containing microtubule singlets, doublets or triplets depending on the cellular and species context (Fig. 1) (Carvalho-Santos et al., 2011). Centrioles duplicate once per cell cycle, where two new centrioles form at the proximal end of the existing pair of centrioles, resulting in inherent age and structural asymmetry. This difference is distinguished by the presence of two sets of appendages, the distal and subdistal appendages, located on the mother (older) centriole (Hall and Hehnly, 2021). Not all species have centrioles that are decorated by appendages (Fig. 1); however, when present, the distal appendages exhibit a nine-fold symmetry, whereas subdistal appendage organization varies based on the species and cell type (Uzbekov and Alieva, 2018). Appendages are closely associated with the ability of an animal cell to form cilia; although there are examples of appendage-less centrioles in *Drosophila* and *Caenorhabditis elegans* that can form specialized sensory cilia, these cilia lack the 9+2 microtubule arrangement (Carvalho-Santos et al., 2011).

In addition to centriolar functions in cilia formation, there are a variety of conserved cellular processes that centrioles are involved in. One such process is cell division, where centrioles recruit PCM proteins and microtubule-nucleating complexes to form the mitotic spindle (Vertii et al., 2016). Centrioles also serve a role in the fertilization of the oocyte (a mature female gamete). In this case, the sperm supplies centrioles to the centriole-deprived oocytes to establish a zygotic centrosome and subsequently initiates embryonic development (Avidor-Reiss et al., 2020). Another function described for centrioles is in membrane trafficking, where the subdistal appendages of the mother centriole have been shown to interact with recycling endosomes and are required for their localization and activity at the centrosome (Hehnly et al., 2012).

Overall, the centrosome plays important conserved roles in most animals. Despite centrosomes and their substructures being highly studied and annotated, a vast amount of our knowledge stems from *in vitro* cell culture and invertebrate *in vivo* studies whereas much less is known about centrosomes and centrioles during early vertebrate and mammalian embryonic development. Understanding how the structure and function of centrosomes varies across animals is especially interesting within the context of a developing embryo, where centrosome structure and function might change as the embryo develops; this has recently been shown to be the case in *C. elegans* embryos, where 88% of embryonic cells lose centrioles during embryogenesis (Kalbfuss and Gönczy, 2023).

In this Review, we discuss aspects of what is known to date about the conserved and divergent features of centrosomes/centrioles during early embryonic development with a comparison to what we know of vertebrate and mammalian embryo systems. We also highlight a subset of processes requiring centrosomes, while also acknowledging the likelihood of additional roles, including centrosomes serving as a central hub for integrating and coordinating signaling pathways, influencing cell cycle regulation, development and DNA damage responses (Arquint et al., 2014). Our focus is to prompt consideration of how centrosomal impacts on signaling might have nuanced variations between animal species owing to subtle alterations in centrosome composition.

## Centrosomes during gametogenesis

Gametogenesis, comprising oogenesis and spermatogenesis, orchestrates the transformation of primordial germ cells into mature female and male gametes. During development, each primordial germ cell harbors a single centrosome, composed of two centrioles in quiescence, that duplicates into two centrosomes housing four centrioles during cell division (Sun and Schatten, 2007). However, the progression towards mature and specialized reproductive cells (the ovum in females and spermatozoa in males) entails a process known as centrosome reduction (Manandhar et al., 2005; Sathananthan et al., 2006). In this section, we systematically explore the existing knowledge surrounding centrosome structures and their associated substructures, dissect the variability in centrosome organization and postulate potential implications for their functional contributions during the dynamic process of gametogenesis across select species.

### Centrosome elimination in oogenesis

**Fig. 2.**
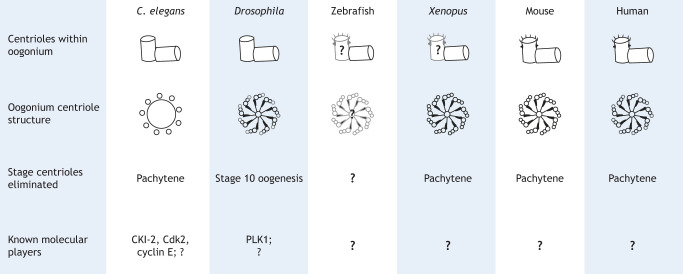
**Centriole elimination during oogenesis across model organisms.** Oogonium centriole composition and structure (not drawn to scale) varies across species. Oogonia from model species contain typical centriole numbers and structures with respect to the species they come from. The timing of oocyte centriole loss is conserved across model species and occurs during the long meiotic prophase, specifically during pachytene in *C. elegans*, *Xenopus*, mice and humans, and the equivalent stage 10 of oogenesis in *Drosophila*. This has not been directly studied in zebrafish, but it is likely that centriole loss is also conserved. Some of the molecular players involved in centriole elimination have been described for *C. elegans* and *Drosophila*, but the molecular mechanisms underlying centriole elimination are yet to be fully understood.

Oogenesis provides an excellent example of the dynamic structural changes a centrosome can undergo. At the beginning of oogenesis, the oogonium of most species have the expected centrosome and centriole numbers and structures (Fig. 2). As oogenesis proceeds, centrosomes and their centrioles are eliminated, resulting in mature ovules lacking centrosomes. The timing of centrosome loss from oocytes is highly conserved, occurring during the long meiotic prophase-I of most known model systems. Specifically, centrioles are eliminated during the pachytene stage in *Caenorhabditis elegans* (Albertson and Thomson, 1993), humans (Manandhar et al., 2005), mice (Szollosi et al., 1972), *Xenopus laevis* (Gard, 1994) and during stage 10 of oogenesis in *Drosophila melanogaster* (Fig. 2) (Dävring and Sunner, 1977). Specific studies have not described the timing of centriole elimination in other model species, such as zebrafish, but it is likely that their centrosomes are also eliminated during prophase-I; a recent study demonstrated the presence of a zygotene-specific oocyte cilium associated with a γ-tubulin-positive centrosome that the cilium was dismantled by the pachytene stage (Mytlis et al., 2022).

There are two strategies for the reduction of centrosomes from female germ cells: elimination or extrusion. The elimination strategy occurs in humans, mice, *Xenopus*, *C. elegans* and *Drosophila*, where centrosomes are eliminated before meiotic divisions (Mikeladze-Dvali et al., 2012; Pimenta-Marques et al., 2016; Sathananthan et al., 2006; Szollosi et al., 1972). By contrast, during extrusion, centrosomes are present during the meiotic division of the female germ cells and are later physically eliminated by polar body extrusion; this has been reported to occur in starfish (Borrego-Pinto et al., 2016) and likely blue mussel (Longo and Anderson, 1969).

Mechanisms of centrosome elimination are generally conserved across species; the centrosome first loses its microtubule nucleating capacity, followed by loss of PCM components, which typically involves diffusion of PCM proteins in the cytosol. Finally, the centrioles are eliminated (reviewed in Manandhar et al., 2005; Schatten, 1994). Although some of the molecular mechanisms of centrosome elimination from invertebrate female germ cells are known, little is known about the molecular players in vertebrates. Studies in *Drosophila* suggest that during centrosome elimination, the downregulation of PCM components is associated with the downregulation of the mitotic kinase polo-like kinase 1 (PLK1). The authors hypothesize that proper PLK1 levels serve to protect against PCM shedding and subsequently centriole loss (Fig. 2) (Pimenta-Marques et al., 2016). In *C. elegans*, centrosome elimination is regulated by the cell cycle checkpoint cyclin E–Cdk complexes, levels of which are controlled by the cyclin-dependent kinase (Cdk) inhibitor *cki-2* (Fig. 2) (Dae and Roy, 2006). This suggests that centrosome elimination requires reduced cyclin E–Cdk complex levels. To our knowledge, literature describing molecular components in oocyte centrosome elimination is limited across most vertebrate species. Recently in mice, the centriole appendage protein outer dense fiber of sperm tails 2 (Odf2), also known as cenexin in humans, was found to localize to microtubule-organizing centers (MTOCs), chromosome centromeres and cytoplasmic vesicles during oocyte meiotic progression, despite the absence of intact centrioles (Xu et al., 2023). This suggests that centriole structural proteins might not be eliminated and are instead reorganized to regulate spindle assembly and positioning after the elimination of centrioles. Thus, further studies are needed to gain a comprehensive understanding of the mechanisms and molecular players involved in centrosome reduction in oocytes, and which centrosome components remain to support meiotic processes.

### Centrosomes during spermatogenesis

**Fig. 3.**
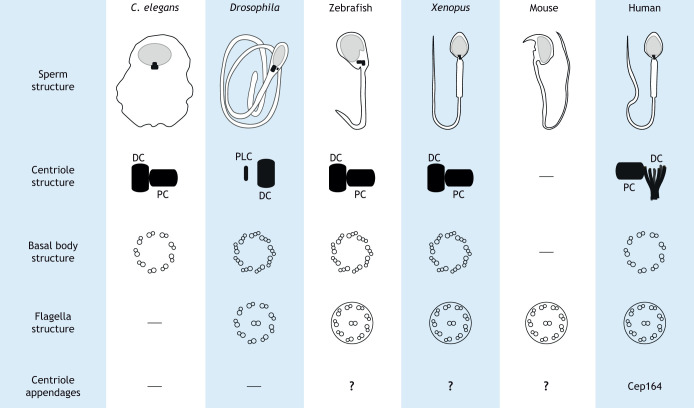
**Divergence in the structure of the sperm and its centrioles across model organisms.** Sperm structure (not drawn to scale) varies across species. Most sperm cells require centrioles to nucleate flagella required for sperm motility. *C. elegans* and mouse sperm are exceptions, where *C. elegans* sperm lack a tail and mouse sperm lacks centrioles. The structure of the sperm centriole pair, basal body and flagella are shown. Whether centriole appendages are present or important in sperm is not yet known, and only one appendage protein, Cep164, has been annotated in human sperm.

Sperm centrioles are essential for several processes, including cell division during spermatogenesis, forming the sperm flagellum (except in *C. elegans*), controlling flagella beating and linking the sperm head to its tail (Avidor-Reiss et al., 2020). Most non-mammalian vertebrates, such as fish and tetrapods, have two canonical barrel-like centrioles in their spermatozoa that are essential post-fertilization (Zhang et al., 2014). *C. elegans* sperm have two centrioles, but their sperm is amoeboid and lacks centriole-templated flagella (Fig. 3) (Singson, 2001). Humans, along with most studied mammals, have spermatozoan centrioles, although only the proximal centriole possesses the canonical structure. The distal centriole has an atypical fan-like structure that is part of the ‘transmission system’ that connects the sperm tail to the head (Fig. 3) (Khanal et al., 2021). This atypical centriole structure reduces microtubule triplets to doublets, which are splayed and flanked by bars made of centriole luminal proteins, such as centrin, Cep44, POC1B and POC5 (Le Guennec et al., 2020; Steib et al., 2020). These rods, along with other structures (e.g. segmented columns and the proximal centriole), move relative to each other during tail beating, forming a dynamic basal complex, which mechanically couples the sperm head and tail (Khanal et al., 2021). This is different from the situation in some invertebrate models, such as *Drosophila*, where the distal centriole resembles a canonical structure, whereas the proximal centriole forms an atypical proximal-centriole-like (PCL) structure (Fig. 3). The PCL resembles a procentriole intermediate that lacks microtubules and is composed of an electron-dense wall with a wide central tube (Avidor-Reiss, 2018). In stark contrast, despite maintaining a 9+2 structured flagellum, spermatozoa from mice and other rodents lack any recognizable centrioles in the early embryo (Fawcett, 1965; Woolley and Fawcett, 1973). This leaves us to ask how the centriole, an essential spermatozoan structure in most animals, became modified and ultimately, dispensable in mice.

One possible explanation for the dispensability of the centriole in rodent spermatozoa that has been proposed is an evolutionary sex cascade theory (Parker, 2014). The cascade is proposed to be driven largely by post-ejaculatory selection that includes sperm competition and female choice that leads to the production of spermatozoa with varying centriole numbers, morphologies and behaviors (Lüpold et al., 2020). One recent study (Khanal et al., 2023 preprint) proposed a potential four-stage centriolar evolutionary cascade impacting sperm in mammals, starting with stage 1, the pre-mammalian centriolar configuration. Here, there is a canonical proximal and distal centriole present in sperm that will be required for early embryogenesis (zygote to blastocyst) where there is no requirement for a female reproductive tract (like in zebrafish). Stage 2 is the mammalian centriolar configuration, where sperm competition within the female reproductive tract evolutionarily selected for sperm with atypical centrioles. Stage 3, characterized by reduced offspring due to miscarriage, exerted selective pressure in ancestral rodents, leading to the evolution of centriole-independent embryonic development. In stage 4, characterized by centriole-independent embryonic development, sperm centrioles became unnecessary, facilitating modifications to proximal and distal centriolar structures, enhancing competitiveness and giving rise to a new sperm neck attachment type (Khanal et al., 2023 preprint). This coincided with the gradual evolution of the luminal centriole scaffold protein FAM161A in rodents and the discovery of a subset of centriole proteins present in sperm necks, suggesting the presence of a highly modified remnant of centrioles (Khanal et al., 2023 preprint).

With general vertebrate ciliogenesis, the mother centriole attaches to the cell membrane through its distal appendages, allowing the cilium to extend into the extracellular space. During spermatogenesis, the structure of the mother or distal centriole can vary across species; for example, in humans and most mammals, no appendages are discernible (Fig. 3). Although they contain the distal appendage protein Cep164, they lack another distal appendage protein, Cep89 (Fishman et al., 2018), necessary for membrane docking of canonical centrioles in cells that form primary cilia (Tanos et al., 2013). Recently, the subdistal appendage protein Cep128 has been shown to be important for flagella formation during spermatogenesis in mice and humans (Zhang et al., 2022), as its loss resulted in defective sperm flagella and infertility. No other studies, to our knowledge, have tested the presence of the centriole appendage proteins in the sperm of other species. Zebrafish sperm might serve as a good model to investigate sperm centriole appendage conservation and function compared to humans as it maintains both of its centrioles (Zhang et al., 2014). These studies can provide information about which appendage proteins are required and which are dispensable for sperm flagella formation and function.

## Functions of centrosomes in early embryonic development

In most species, sperm centrioles, devoid of PCM, recruit maternal PCM proteins upon reaching the egg during fertilization, forming a functional centrosome for embryo development (Schatten and Sun, 2011). This initial zygotic centrosome establishes a substantial microtubule aster for female and male pronuclei migration and congregation. Subsequently, it duplicates to create the two spindle poles vital for the first zygotic cell division. However, in mice, owing to the absence of centrioles in both oocytes and sperm, the first zygotic centrosome necessitates *de novo* assembly (Gueth-Hallonet et al., 1993; Xiao et al., 2021). Recent studies in non-rodent mammalian systems have revealed a potentially similar scenario (reviewed in Uzbekov et al., 2023). Here, we explore the distinctive mechanisms employed by early mammalian embryos in centrosome establishment.

### The centrioles and mother centriolar appendages

**Fig. 4.**
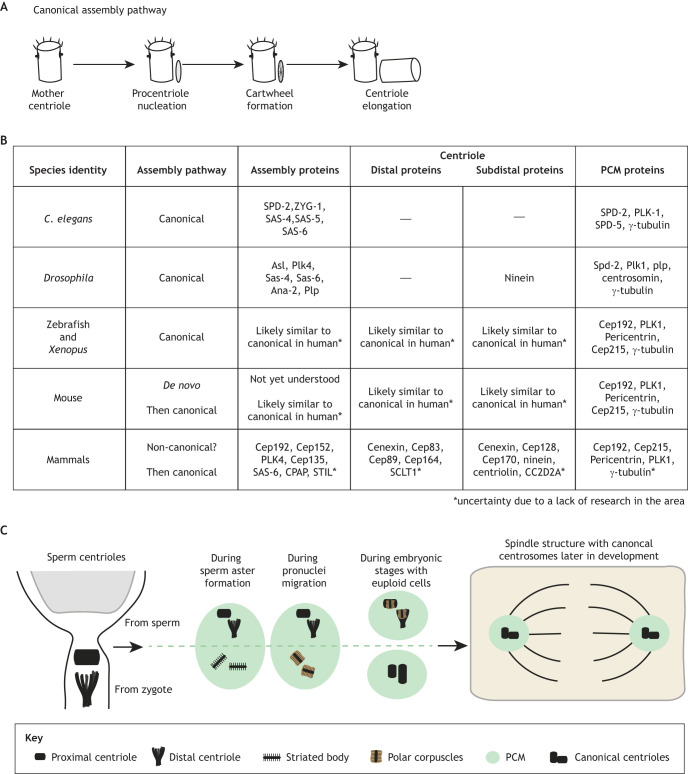
**Centrosome assembly across model species.** (A) Representation of the canonical centriole assembly pathway. (B) Centrioles are assembled via the canonical pathway in most embryonic species except for mammalian embryos, where mouse centriole assembly occurs first through a *de novo* pathway, whereas non-rodent mammals are suspected to form their centrioles through a non-canonical pathway. This is followed by a canonical (templated) pathway for centriole assembly later in development. Proteins involved in the assembly of centrioles, distal appendages, subdistal appendages and the PCM are compared across species. Dashes represent the absence of a structure. (C) A model depicting the change in centriole structure throughout human and bovine development. In most non-rodent mammals, sperm have a proximal centriole (PC) and an atypical distal centriole (DC). Sperm atypical centrioles and polar corpuscles (also atypical centrioles) are found in the zygote and act to recruit pericentriolar material (PCM), form centrosomes, and serve as a platform for the assembly and duplication of centrioles.

In invertebrates and non-mammals, centriole assembly in early embryos and dividing somatic cells relies on a conserved set of proteins that undergo template-dependent centriole assembly. These proteins are highly conserved and shared among *C. elegans* (Carvalho-Santos et al., 2010; Pelletier et al., 2006), *Drosophila* (Azimzadeh and Marshall, 2010; Ito et al., 2019) and humans (Sonnen et al., 2012; Tsou and Stearns, 2006) (Fig. 4A,B). Recent intriguing developments lie in mammalian centriole assembly during early embryogenesis. Previous studies have supported the model that human sperm provides only the proximal centriole, which possesses a typical cylinder-like shape (Fishman et al., 2018; Manandhar et al., 2000). A recent report, however, has identified that the sperm also contributes the distal centriole, which exhibits an atypical structure (Uzbekov et al., 2023) (Fig. 4C). The distal centriole contains centriole-specific proteins, including centrin, POC5, FAM161A and POC1B. During fertilization, this atypical centriole can recruit PCM, forming a centrosome that establishes a large microtubule aster, facilitating the union of the male and female pronuclei (Fishman et al., 2018). Within this nucleating complex, two newly identified structures, referred to as striated bodies (Uzbekov et al., 2023), are hypothesized to be precursors of atypical centrioles known as polar corpuscles. During this stage, there are four structures – two polar corpuscles, a modified proximal centriole and a distal centriole located at the junction of the pronuclei. These structures are suggested to be necessary for redistributing chromosomes within the pronuclei for accurate segregation (Cavazza et al., 2021).

A variety of combinations of canonical and atypical centrioles and their intermediates are present during the cleavage stage divisions in the mammalian embryo. For example, both the distal and proximal centrioles combine to form a single mitotic aster within the first spindle that forms. A pair of polar corpuscles goes on to form the second aster of the spindle (Uzbekov et al., 2023). Notably, the mother and daughter centrioles (i.e. distal and proximal centrioles) remain together at one pole and, as cell divisions progress, both centrioles start to adopt atypical appearances. It is only at the third cleavage of the embryo that the two canonical centrioles emerge. At this stage, the mother centriole seems to lack subdistal or distal appendages.

In most animal species, including *C. elegans*, *Drosophila* and vertebrate and mammalian somatic cells, centriole formation follows a templated process, with daughter centrioles assembling at the proximal end of mother centrioles (Fig. 4A). This process involves procentriole nucleation, cartwheel formation, then centriole elongation (reviewed in Avidor-Reiss and Gopalakrishnan, 2013). Notably, in mouse embryos, *de novo* centriole assembly is observed at the 32-cell stage, becoming more consistent by 64–128 cells, marking the transition to templated centriole duplication (Abumuslimov et al., 1994; Gueth-Hallonet et al., 1993; Howe and FitzHarris, 2013; Schuh and Ellenberg, 2007). Recent reports indicate that centriole appendage proteins might progressively accumulate at centrioles during pre-implantation to gastrulation (Xiao et al., 2021), potentially playing a role in organogenesis and enabling cells to initiate cilia formation. Specific examples of this are studies investigating the loss of two different sub-distal appendage proteins, Odf2 (Ishikawa et al., 2005; Salmon et al., 2006) and Cep128 (Mönnich et al., 2018). In murine somatic cells, Odf2 is a protein required for the structural integrity of the appendages (Ishikawa et al., 2005), and its loss causes murine preimplantation lethality of the developing blastocyte (Salmon et al., 2006). Similarly, Cep128 loss in zebrafish results in organogenesis defects (Mönnich et al., 2018). The dynamic centriole combinations and gradual appendage protein accumulation in early embryogenesis offer insights into centriole diversity and potential roles in organogenesis, as evidenced by studies on Odf2 and Cep128.

Whereas *de novo* centriole assembly is thought to be mostly associated with rodent embryogenesis, aspects of *de novo* centriole assembly likely also occur in non-rodent mammalian embryogenesis due to the identified non-canonical centriole structures modeled in Fig. 4C (Avidor-Reiss and Uzbekov, 2023). However, the molecular mechanisms responsible for this type of assembly are far less understood. This raises questions about when appendage proteins are present and when their structures emerge during embryonic development. Currently, our understanding of appendage structure during early vertebrate mammalian development is limited, and it remains unclear when appendage formation initiates and how it impacts embryonic function. Nonetheless, it appears that appendage formation occurs after canonical or template-dependent centriole assembly.

Meanwhile, in other organisms, canonical centrioles are crucial for early embryogenesis. In *C. elegans*, disruption of canonical centrioles results in developmental arrest (Delattre and Gönczy, 2004) and in *Drosophila*, this leads to embryonic lethality (Leidel and Gönczy, 2003; Megraw et al., 1999; Stevens et al., 2007; Varmark et al., 2007). Conversely, in mammalian embryos, the absence of canonical centrioles during initial divisions prompts reliance on atypical centriole structures for early developmental processes, especially when cilia are not yet formed. Notably, atypical centriole structures have been observed in bovine (Uzbekov et al., 2023) and human embryos (Sathananthan et al., 1991). In summary, the dynamics of centriole structures in early vertebrate embryos remain a relatively unexplored area, offering potential insights into spindle formation and centriole generation crucial for later cilia development.

### Pericentriolar material

PCM assembly has been extensively studied during somatic cell divisions and in invertebrate embryogenesis (reviewed in Gönczy and Hatzopoulos, 2019; Lee et al., 2021). As cells progress through the cell cycle, cytosolic PCM proteins accumulate at the centrosome, where they will ultimately be assembled or translated, contributing to the expansion of the interphase PCM layer. A core set of proteins involved in PCM assembly and expansion, including Cep192, PLK1, pericentrin, Cep215 (also known as CDK5RAP2) and γ-tubulin, is conserved across species from *Drosophila* to humans (Fig. 4B) (Colicino and Hehnly, 2018; Conduit et al., 2010, 2014; Decker et al., 2011; Dictenberg et al., 1998; Lee and Rhee, 2011; Wong et al., 2022; Woodruff et al., 2014). However, in *C. elegans*, a smaller set of proteins, including SPD-2, PLK-1, SPD-5 and γ-tubulin, orchestrates PCM assembly (Fig. 4B). Notably, SPD-5, although lacking sequence homology, functions as a potential counterpart to Cep215, guiding PCM component recruitment (Hamill et al., 2002; Woodruff et al., 2015, 2017). A recent focused ion beam scanning electron microscopy (FIB-SEM) study revealed potential regulation of *C. elegans* PCM size by the endoplasmic reticulum (ER), with images showing ER enveloping the centrosome, with its size potentially determined by the surrounding ER membrane (Maheshwari et al., 2023). This finding introduces a novel aspect to known PCM organization mechanisms, warranting investigation in vertebrate and mammalian systems.

Although many zygotic PCM proteins are traditionally considered maternally inherited and used for the first several rounds of division (Schatten and Sun, 2011), a potential exception arises in human sperm. A recent study has demonstrated that certain PCM proteins, specifically γ-tubulin and pericentrin, are noted as being paternally inherited (Amargant et al., 2021). This paternal contribution is underscored by the absence of pericentrin in human oocytes. The sperm contribution of pericentrin and γ-tubulin is likely not enough to support multiple rounds of spindle formation, suggesting an additional hypothesis that pericentrin and γ-tubulin might be maternally expressed after fertilization. Indeed, studies during zebrafish embryogenesis suggest that PCM expansion involves co-translational targeting mechanisms, delivering pericentrin transcripts to the centrosome where they are ultimately translated during early development (Sepulveda et al., 2018). Recent studies spotlight the centriole satellite protein, cfap53, as a key player in the formation of the initial MTOC during zebrafish embryo development (Willekers et al., 2022). Homozygous cfap53 mutations result in impaired γ-tubulin localization and one-cell stage arrest. This implies that certain centriole satellite proteins, possibly co-expressed with γ-tubulin maternally, play a vital role in establishing the first zygotic PCM in vertebrate embryos.

Centrosome size, which is mostly governed by the recruitment and organization of PCM, might play a critical role during embryogenesis. In zebrafish embryos, we found that the size and organization of PCM between the two spindle poles closely correlate with the size of the embryonic cell, a process regulated by PLK1 (Rathbun et al., 2020). Our study revealed that mitotic centrosomes scale proportionally with changes in cell size. Furthermore, we observed that the centrosomes across the spindle exhibited asymmetry in size. This asymmetry did not necessarily result from increased PCM recruitment to one pole over the other; instead, we propose that one pole might have more tightly packed PCM components in comparison to the other. However, both poles became more uniform in size at around the 512-cell stage (Rathbun et al., 2020). Considering our previous discussions regarding the timing of canonical centriole formation in mammalian models (Fig. 4), and potentially in vertebrate models like zebrafish, where this timing remains uncertain, we suggest a scenario in which centriole formation might serve as the foundation for the signaling required for PCM packing that we refer to as cohesion. In line with this, studies have shown that an appendage protein, ODF2, interacts with PLK1, playing a role in regulating PCM cohesion (Aljiboury et al., 2022; Soung et al., 2006). However, at early embryonic stages, vertebrate and mammalian embryos likely lack appendage proteins (Uzbekov et al., 2023; Xiao et al., 2021). This raises the hypothesis that the onset of appendage protein expression and the appearance of centrioles contribute to PCM concentration as embryos progress through development. To address this idea, a clearer characterization of PCM dynamics and appendage protein expression and organization during early vertebrate and mammalian embryo development is essential.

## Cilia during embryonic development

**Fig. 5.**
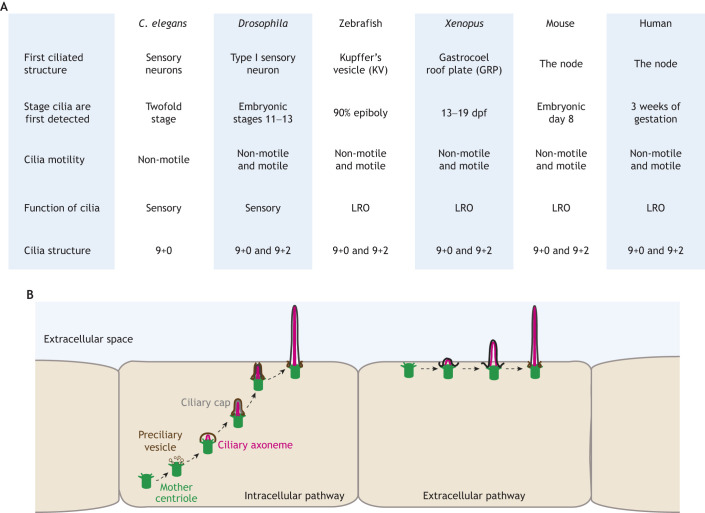
**Cilia during embryonic development.** (A) Embryonic stages at which cilia are first detected, and their motility and structure across select species. The first ciliated structure in *C. elegans* and *Drosophila* are in sensory neurons, which arise later in development compared to in most vertebrate species, which develop their ciliated cells early during left-right organizer (LRO) formation. Both *C. elegans* and *Drosophila* cilia have a 9+0 structure. The LRO from vertebrate species contains both motile and non-motile cilia with 9+2 and 9+0 structures. (B) The intracellular cilia assembly pathway begins with the docking of small vesicles (preciliary vesicles) to the distal appendages of the mother centriole that then form the ciliary vesicles, which later give rise to the ciliary sheath. Ciliary vesicle formation is associated with the removal of the ciliary cap, a protein structure that blocks cilia formation. Ciliary membrane and axoneme elongation then occur within the intracellular matrix of the cell. The basal body, ciliary sheath and axoneme complex then dock and fuse to the plasma membrane, after which cilia can extend into extracellular space. In the extracellular pathway, the mature mother centriole first migrates towards the cell surface, where it attaches to the plasma membrane through its appendages, followed by ciliary axoneme formation and extension into the extracellular space.

Cilia are hair-like, microtubule-based structures that extend outward from a specialized structure known as the basal body, which is derived from the mother centriole (Fig. 5) (Avasthi and Marshall, 2012). These structures can exhibit motility and are sometimes referred to as flagella or can remain non-motile, such as sensory or primary cilia. The axoneme is the core microtubule-based structural component of a cilium that is responsible for its movement in motile cilia or for providing structural support in non-motile cilia. Motile cilia feature nine microtubule doublets with a central pair of microtubules (referred to as 9+2), whereas non-motile cilia lack this central pair (9+0; Fig. 1) (Wheatley et al., 1996). It is worth noting that studies have demonstrated that both primary/non-motile and motile cilia can exhibit configurations of 9+0 and 9+2 (Satir and Christensen, 2007). In this section, we will discuss the current knowledge regarding when cilia first appear during embryonic development in various species and the functions they serve.

In *C. elegans*, none of the cilia are motile, and the only ciliated cells are sensory neurons, which develop at the latest stages of embryogenesis (Nechipurenko et al., 2017). These cilia exhibit a 9+0 axonemal structure typical of non-motile cilia (Figs 1 and 5). Similarly, most *Drosophila* cells lack cilia, except for type-I sensory neurons of the peripheral nervous system and sperm cells (Jana et al., 2016). In *Drosophila* sperm, the cilia/flagella are motile with a 9+2 axoneme structure (Figs 1 and 5) (Zur Lage et al., 2019). All the sensory cilia of *Drosophila* have a 9+0 structure; however, their motility differs depending on the neuron subtype. For instance, external sensory neuron cilia are non-motile, whereas chordotonal neuron cilia are motile, reflecting their function in sensory mechanotransduction and frequency tuning (Zur Lage et al., 2019).

Cilia in vertebrate systems are formed earlier during embryonic development than in *C. elegans* and *Drosophila*. In most vertebrates, cilia formation is first recorded during or before the development of the left-right organizer (LRO), a fluid-filled tissue responsible for left-right patterning in the early embryo (Capdevila et al., 2000). LRO cells can have both motile and non-motile cilia with a 9+2 or 9+0 axonemal structure based on the species, but most vertebrate systems have both (Fig. 5A) (Dasgupta and Amack, 2016; Djenoune et al., 2023; Hyland and Brody, 2022; Okabe et al., 2008; Sulik et al., 1994). Motile LRO cilia generate a directional fluid flow to break bilateral symmetry and regulate the placement of visceral organs. Non-motile LRO cilia make the symmetry-breaking process more robust by responding to the flow generated by motile cilia (Djenoune et al., 2023; Katoh et al., 2023). When LRO cilia are disrupted, nodal flow gets disrupted, resulting in left-right asymmetry defects (Djenoune et al., 2023; Katoh et al., 2023). In human embryos, the LRO (node) arises at 3 weeks of gestation and is the first organ in which cilia are detected (Hyland and Brody, 2022). However, the formation of cilia during human node formation is not fully annotated. It is possible that in humans, cilia are formed before the development of the node, as is the case for zebrafish embryos (Aljiboury et al., 2023). Like in humans, the zebrafish LRO (Kupffer's vesicle, KV) is the first annotated ciliated organ in the developing embryo (Essner et al., 2005). From our recent studies, we know that cilia are formed prior to KV lumen development (Aljiboury et al., 2023). We found that ∼33% of KV precursor cells had cilia at the 90% epiboly stage, suggesting that cilia formation is a precursor to LRO formation. In mice, cilia are first detected at embryonic day (E)5.5 in less than 1% of the epiblast cells, which increases to ∼32.7% by E6.0, and eventually, all epiblast-derived cells become ciliated at E8, including the node, which is fully formed at this stage (Bangs et al., 2015). As is the case in vertebrate models discussed so far, the first ciliated organ formed in *Xenopus* is the LRO, also known as the gastrocoel roof plate (GRP), between stages 13 and 19 (1dpf) of embryonic development (Blum et al., 2014; Walentek and Quigley, 2017). In all these species, cilia in the LRO are essential for laterality determination, cardiac formation and the placement of visceral organs. Defects in cilia in the LRO have been associated with inversed organ placements and congenital heart diseases (Forrest et al., 2022).

All cilia are nucleated from basal bodies (mature mother centrioles) and can be formed by either an intracellular or extracellular pathway (Fig. 5B). The intracellular pathway cilia form within the intracellular space of the cell, migrate to the apical membrane and then extend into the extracellular space. In the extracellular pathway, the mature mother centriole first migrates from a centrally located position to an apical or forming apical membrane and attaches to the cell surface, where ciliary axoneme formation and extension into the extracellular space can occur (Sorokin, 1962). Cells with 9+0 axoneme structures, such as fibroblasts, mesenchymal cells, photoreceptors and retinal epithelial cells, are typically associated with the intracellular pathway, whereas multi-ciliated cells or cells with primary cilia might use the extracellular mechanism (Zhao et al., 2023). Most studies that identified molecular mechanisms for intracellular primary cilia formation relied on *in vitro* cell culture (Knödler et al., 2010; Kuhns et al., 2019; Westlake et al., 2011; a recent review is provided by Zhao et al., 2023). However, our recent study identified that the zebrafish LRO also uses an intracellular mechanism for motile cilia formation that involves the small GTPases Rab11 and Rab35 (Aljiboury et al., 2023). What is yet to be resolved, however, is what determines when a cell uses intracellular versus extracellular mechanisms for cilia formation during development. One idea we propose is that this could be dictated by developmental contexts; for instance, cells that coordinate cilia formation and *de novo* lumen formation might employ an intracellular mechanism of cilia formation to assist with membrane remodeling, facilitating lumen opening. In contrast, cells that already have a designated extracellular space can potentially use extracellular mechanisms for cilia formation.

## Concluding remarks

The centrosome (and its associated cilium) is a versatile organelle that is involved in a host of cellular and developmental processes. While *in vitro* and invertebrate models have provided insights into their functions, vertebrate studies are equally valuable. For instance, in mice, unlike in invertebrate models, centrioles appear to be dispensable for fertilization and early zygotic divisions (Avidor-Reiss and Uzbekov, 2023), highlighting the relevance of vertebrate investigations. Furthermore, in vertebrate models, centrosomes, which acquire distal and subdistal appendages, exhibit divergent centrosome-related processes compared to their invertebrate counterparts, potentially affecting the initial cilia assembly pathways. Vertebrates also possess complex ciliary structures that play pivotal roles in various physiological processes including organ development, tissue homeostasis and signal transduction (extensively reviewed in Anvarian et al., 2019; Mill et al., 2023; Pazour et al., 2020). Thus, we argue that studying the role of the centrosome and cilia in vertebrate models, such as mice, zebrafish and *Xenopus*, at early stages of development is crucial for comprehending how ciliary dysfunction contributes to human diseases like ciliopathies, developmental disorders and infertility. These studies will also give insights into the temporal requirement for centrioles and their substructures in embryogenesis and beyond, as well as for their role in development.
